# Influence of age and gender on sex steroid receptors in rat masticatory muscles

**DOI:** 10.1038/s41598-019-54774-y

**Published:** 2019-12-05

**Authors:** Alessandra Pucci Mantelli Galhardo, Márcio Katsuyoshi Mukai, Matsuyoshi Mori, Katia Candido Carvalho, Maria Cândida Pinheiro Baracat, Manuel de Jesus Simões, José Maria Soares, Edmund Chada Baracat

**Affiliations:** 1 Departamento de Obstetrícia e Ginecologia do Hospital das Clínicas da Faculdade de Medicina da Universidade de São Paulo, São Paulo, Brasil; 2 Departamento de Prótese da Faculdade de Odontologia da Universidade de São Paulo, São Paulo, Brasil; 3 Departamento de Morfologia e Genética da Universidade Federal de São Paulo, São Paulo, Brasil

**Keywords:** Skeletal muscle, Mandibular muscles

## Abstract

The temporomandibular muscle dysfunction is characterized by myofascial pain and is more prevalent in women of reproductive age. Sex steroid hormones are hypothetically involved in the dysfunction, but few are the studies of steroid receptors in masticatory and mastication-related muscles. Our aim was to determine estrogen and testosterone receptor expression in rat masticatory and mastication-related muscles within the context of age and gender. Twelve rats were equally divided into four groups: (a) 10-month-old females; (b) 10-month-old males; (c) 24-month-old females; and (d) 24-month-old males. Euthanasia of the females was performed in the proestrous phase (vaginal smears) and the masticatory and accessory muscles were removed for immunohistochemical analysis. Statistical analysis was performed with ANOVA and the Tukey test. Estrogen receptor expression was similarly low in all muscles and groups. Testosterone receptor expression in the Masseter muscle of the 24-month-old male rats was higher than that in the other groups and significantly superior to its expression in the Posterior Digastric muscle. In short, testosterone receptor expression was highest in old male rats. If we generalize to humans, this fact could indicate age- and sex-related hormonal influence on temporomandibular muscle dysfunction. Further studies, however, are necessary to strengthen this hypothesis.

## Introduction

Myofascial pain, a characteristic of temporomandibular muscle dysfunction (TMD), is possibly influenced by sex hormones, given that it is more prevalent in women of reproductive age^1–7^. A few researchers have suggested that the fluctuation of sex steroids during the menstrual cycle is what causes the disturbance^8–10^. However, such a symptomatic pain is also felt by contraceptive hormone users, i.e., the pain occurs in the absence of hormone fluctuations, but the hormones are still present^11^.

Plausibility of estrogen action on myofascial pain is sustained by evidence of the presence of estrogen receptors in the masticatory muscles of female^12^ but not male baboons^13^. Besides, these receptors are highly expressed in the nervous fibers innervating the female rat temporalis muscle and they can modulate the nociception of the muscle^5^. Another hypothesis relates androgens to musculature since they are found in a number of muscles, including those connected with mastication^14–17^. It should be mentioned that testosterone interferes in the postnatal evolution of trigeminal nociception and in the development of the reflex response of the rat Digastric muscle, an accessory muscle of mastication, given the presence of androgen receptors in the motor neurons of the mandibular muscles^18^. Testosterone is likely a factor of protection against masticatory dysfunction.

The rat is considered a good model for many researchers because of the similarity between the human and the rat articulatory and muscular structures of the temporomandibular joint and the low costs relative to other models^19,20^. The masticatory muscles are the Masseter, Temporal, Medial Pterygoid, and Lateral Pterygoid. The latter is a muscle of small dimensions whose origin and point of insertion hinder removal without laceration^21^. There are other muscles which aid the aforementioned dynamic muscles but have no direct impact on the teeth and they are called accessory muscles. The representative of this group is the Digastric (Anterior and Posterior) muscle. The main difference between the human and rat temporomandibular articulation is the biomechanics of movement and the type of incident load resulting from the different nutritional diets among species, albeit a static load is essential for the growth and maintenance of the temporomandibular joint^19–22^. Therefore, physiological experiments have some limitations. Nonetheless, the protein steroid receptors in the muscles are closely comparable to those in humans. In general, it is suggested that a 10-month-old rat corresponds to a 25-year-old human, that is, a human at the peak age of the reproductive phase, and a 24-month-old rat to a middle-aged 60-year-old human^23–25^.

The level variations of sex hormones and their receptors during the menstrual cycle may affect women’s neuromuscular performance and their risk of sustaining musculoskeletal injury^26^. Age is another potential factor affecting the muscles and other organs^27^. In fact, some authors applied testosterone for attenuation of the expected age-related decline in the physical performance of healthy men aged 60 years or older^28,29^, because testosterone might have a protective action on male muscles. In women, the sex steroid receptor may affect muscular performance and thus be the reason for the higher prevalence of muscle TMD in them. In other words, sex steroids acting through their receptors are suspect of playing a role in TMD. However, information about the number of estrogen and testosterone receptors in masticatory muscles, as well as the influence of gender and age on receptor expression, is scarce. Our study, therefore, aimed to analyze estrogen and testosterone receptor expression in rat masticatory and mastication -related muscles and the differences entailed by gender and age.

## Materials and Methods

This study was approved by the Ethics Committee on the Use of Animals of the Instituto de Ciências Biomédicas (ICB), Universidade de São Paulo (USP) and all procedures involving animals in this study were in accordance with the ethical standards of the institution.

### Animals

Twelve Wistar rats (*Rattusnorvegicus*), equally divided into ten-month olds and 24-month olds, as well as into females and males, were sourced from the animal laboratory of ICB-USP. They were fed the AIN-93G rodent diet *ad libitum* as recommended by the American Institute of Nutrition (Rhoster^®^) and kept in a temperature-controlled (23 °C) room with light-dark cycles (LD:12 h/12 h) from birth until they reached an appropriate age.

The animals were raised to be used in this study alone; they were healthy and exhibited no pathologies. Had they developed a pathology or lost a great deal of weight as they aged, they would have been disposed of. However, no such adversities happened. Furthermore, if a muscle was severely lacerated or damaged during sample removal, it would not be included in the analysis. This happened to the Lateral Pterygoid muscle.

Divided according to age and gender as specified above, the 12 animals comprised four groups as follows: (a) three 10-month-old females; (b) three 10 -month-old males; (c) three 24-month-old females; and (d) three 24-month-old males.

### Estrous cycle analysis

The rat estrous cycle consists of the diestrus I, diestrus II, estrus, and proestrus phases, each lasting an average of 24 hours. A complete cycle, therefore, takes approximately 4 days. These phases were determined by analyzing the cell types present in the vaginal secretions of the females, namely many leukocytes, few spindle cells, some epithelial cells (in diestrus), rounded cells, dispersed or pooled polynucleate cells (in proestrus), and cells resembling “dry leaves” (in estrus)^30,31^. The rats had their cycles followed daily for 2 weeks to ensure cycle regularity, i.e., 4-day cycles, a characteristic of the younger animals (10 months) in our study.

The older females (24 months) presented an irregular estrous cycle in which a phase lasted for 4 to 5 days or in which the following sequence was absent: proestrus, estrus, diestrus I, and diestrus II. Nevertheless, we included for analysis only the females which had at least one proestrous phase.

### Euthanasia

The females were euthanized in proestrus and the males at a corresponding age. Prior to sacrifice, the female rats were intraperitoneally anesthetized with 15 mg/kg Xylazine (Rompun^®^; Bayer, Brazil) and 30 mg/kg Ketamine (Ketalar^®^; Pfizer, Brazil)^32^ for the removal of the masticatory muscles.

### Samples

The Masseter, Temporal, Medial Pterygoid (masticatory), and Digastric muscles were removed and immersed in 10% buffered formaldehyde for 48 hours. They were then immersed in 70% alcohol until inclusion in tissue blocks for the subsequent mounting of silanized slides for immunohistochemical analysis. The Lateral Pterigoyd muscles were excluded from the samples due to intense maceration in the removal process rendering them inadequate for analysis.

Once embedded, the tissues were sectioned at 3-µm intervals, 5 slides for each animal. Two cuts were utilized for morphological analysis (hematoxylin-eosin) undertaken with a light microscope (AxioLab, Carl Zeiss^®^) coupled with high-resolution imaging equipment (AxioCam-MCR, Carl Zeiss^®^). Images were transmitted to a computer via a Windows XP^®^ operational system and the Axio Vision Rel 4.2 (Carl Zeiss^®^) software. Measurements were taken in 10 randomly chosen microscopic fields and examined by two researchers (MJS and MCPB) independently, who were blinded to the groups and the tissues.

### Immunohistochemical reactions

All muscle samples were tested using rabbit polyclonal anti-estrogen receptor alpha antibody, (orb13402) (Biorbyt^®^, United Kingdom), dilution 1:800; rabbit polyclonal anti-estrogen receptor beta, chIP grade (ab3577), (ABCAM^®^, United States), dilution 1:800; and rabbit polyclonal anti-androgen receptor antibody, chIP grade (ab74272), (ABCAM^®^, United States), dilution 1:200.

### Reaction controls

Prostate and breast sample tissues were used as controls; the former for androgen receptors and the latter for estrogen receptors. Bovine serum albumin was substituted for the primary antibody and used as a negative control. Other negative controls were employed using non-specific goat antibodies with the same concentration as that of the primary antibody for each immunohistochemical reaction (estrogen or testosterone receptors).

Striated muscle cells were scored negative in the absence of immunopositive cells. The total score reflected the degree of immunostaining intensity as specified by a method modified from Panzan *et al*.^33^.

The percentage of immunopositive cells was estimated based on 10 representative fields. Immunostaining intensity was scored negative (0), very slight (1), weak (2), moderate (3), or strong/intense (4). Two experienced observers blinded to the purpose of the slides made all of the assessments. After completion of the study, the same observers (MJS and MCPB) reexamined the slides to ensure reproducibility of the semiquantitative assessments.

### Statistical analyses

The sample was calculated aiming at a difference of at least 2 standard deviations (SDs) in the number of the muscular receptors of the animals between the peak of the younger and the older animals. The power level was set at 80%^34^.

The data are expressed as mean ± standard deviation (M ± SD). The statistical analyses were performed with GraphPad Prism 5.0^®^ (GraphPad Software Inc.) software. Data distribution was analyzed using the Kolmogorov-Smirnov test. Afterwards, ANOVA and the Tukey post hoc test were employed to analyze the immunohistochemical data. A significance level (α) of 5% was adopted for every test; the p value was calculated and is shown for every statistic.

## Results

Regardless of age and gender, estrogen receptor expression was similarly low in all muscles and groups.

**Figure 1:**
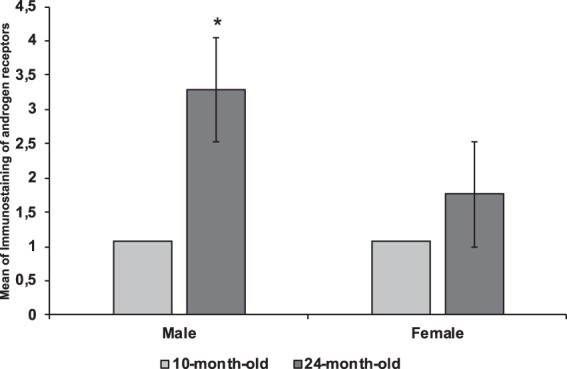
Graphic representation of testosterone receptor expression means in the Masseter muscle. Each column represents a mean and each bar, the standard deviation from the mean. *p < 0.01: 24-month-old male rat group compared to 10-month-old male rat group and the 10- and 24-month-old female rat groups (ANOVA and the Tukey post hoc test with statistically significant results).

**Figure 2:**
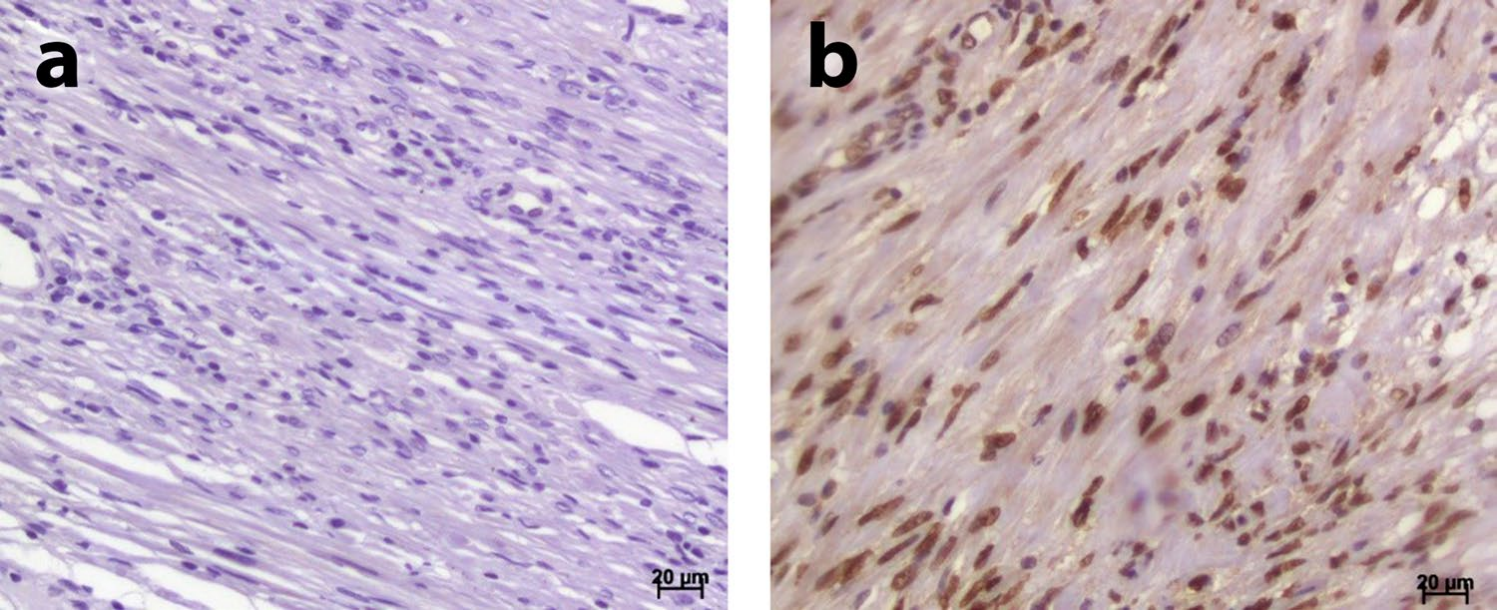
(**a**)Negative control for androgen; (**b**) Positive control for androgen.

**Figure 3:**
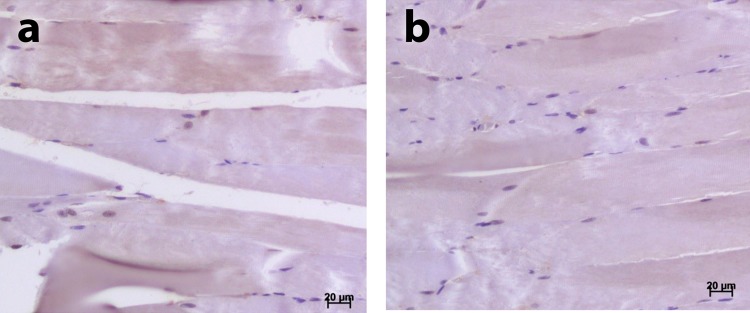
(**a**) Masseter muscle from a 10-month-old male rat; (**b**) Masseter muscle from a 10-month-old female rat.

**Figure 4:**
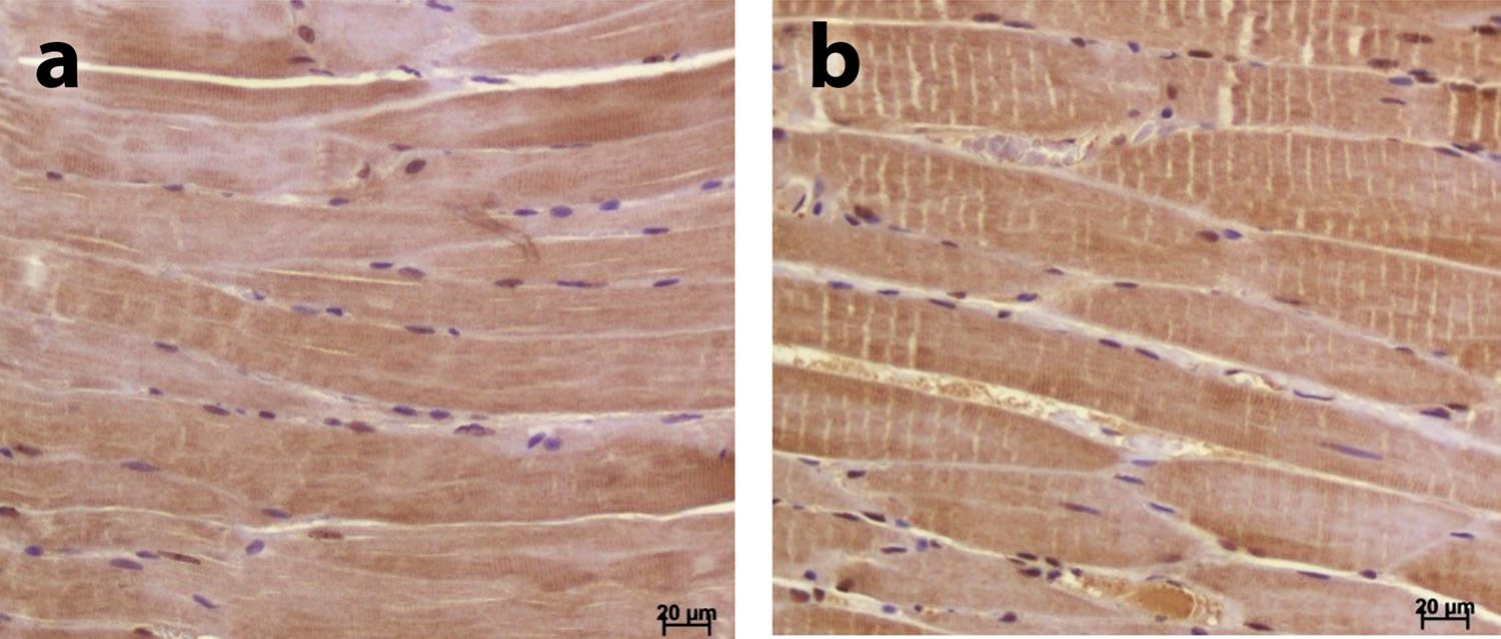
(**a**) Masseter muscle from a 24-month-old male rat; (**b**) Masseter muscle from a 24-month-old female rat. Results are presented as mean and standard deviation from the mean. Age 10 means 10-month-old animals. Age 24 means 24-month-old animals. M1 means male 1. M2 means male 2. M3 means male 3. F1 means female 1. F2 means female 2. F3 means female 3. (**a**) Significant difference (p < 0.01) between testosterone receptor expression in the Masseter muscle of this group and that of the other groups (ANOVA test followed by the post hoc Tukey test); (**b**) significant difference (p < 0.05) in testosterone receptor expression between the Masseter muscle and the Posterior Digastric accessory muscle in this group (ANOVA test followed by the post hoc Tukey test).

Table 1 and Figs. 1–4 display the testosterone receptor results. Testosterone receptor expression was highest in the Masseter muscle of 24-month-old males (3.28 ± 0.39) and the difference between expression in this group and that of the other groups (10-month-old females: 1.07 ± 0.40; 24-month-old females: 1.76 ± 0.67; and 10-month-old males: 1.08 ± 0.39) was significant (p < 0.01). Still in the group of 24-month-old males, testosterone expression in the Masseter muscle was higher than in the Posterior Digastric accessory muscle (0.66 ± 0.45), with no significant differences being found among the other muscles (Temporal, Medial Pterygoid and Anterior Digastric). In the groups other than that of the 24-month-old males, testosterone was similarly expressed in the masticatory muscles.

**Table 1:** Immunostaining of testosterone receptors in the Masseter, Temporalis, Medial Pterygoid, and Digastric (Anterior and Posterior) muscles.

Age (months)	Gender	Masticatory muscles			Accessory muscles	
		Masseter	Temporalis	Medial Pterigoyd	Digastric	
					Anterior	Posterior
10	M1	0.95 ± 0.24	1.08 ± 0.32	1.24 ± 0.45	1.15 ± 0.34	0.99 ± 0.23
	M2	1.31 ± 0.46	0.78 ± 0.44	1.04 ± 0.15	1.05 ± 0.54	1.56 ± 0.43
	M3	0.99 ± 0.48	1.62 ± 0.55	1.16 ± 0.65	1.18 ± 0.12	1.99 ± 0.29
	Mean	1.08 ± 0.39	1.16 ± 0.43	1.15 ± 0.41	1.13 ± 0.33	1.51 ± 0.31
	F1	0.85 ± 0.33	1.18 ± 0.51	1.33 ± 0.45	0.95 ± 0.44	1.43 ± 0.38
	F2	1.21 ± 0.36	0.96 ± 0.62	1.44 ± 0.33	1.56 ± 0.53	0.92 ± 0.43
	F3	1.14 ± 0.53	1.02 ± 0.45	1.45 ± 0.55	0.96 ± 0.83	1.49 ± 0.39
	Mean	1.07 ± 0.40	1.05 ± 0.52	1.41 ± 0.44	1.16 ± 0.6	1.28 ± 0.4
24	M1	3.05 ± 0.34	1.29 ± 0.32	1.15 ± 0.39	1.58 ± 0.32	0.22 ± 0.37
	M2	3.31 ± 0.56	0.66 ± 0.29	0.99 ± 0.54	1.43 ± 0.67	1.28 ± 0.56
	M3	3.49 ± 0.28	1.72 ± 0.23	0.89 ± 0.36	1.48 ± 0.65	0.49 ± 0.43
	Mean	3.28 ± 0.39^a,b^	1.22 ± 0.28	1.01 ± 0.43	1.49 ± 0.55	0.66 ± 0.45
	F1	1.11 ± 0.72	1.88 ± 0.56	2.45 ± 0.80	0.78 ± 0.44	1.84 ± 0.67
	F2	1.56 ± 0.44	2.22 ± 0.45	1.99 ± 0.37	2.31 ± 0.62	1.78 ± 0.44
	F3	2.62 ± 0.87	0.76 ± 0.43	2.34 ± 0.76	2.34 ± 0.66	1.72 ± 0.59
	Mean	1.76 ± 0.67	1.62 ± 0.48	2.26 ± 0.64	1.81 ± 0.57	1.78 ± 0.56

## Discussion

Temporomandibular dysfunctions are related to articulatory and muscular changes, which may result in pain and mastication difficulties^1–7^. The muscles involved in mastication are highly influential in the genesis of the dysfunction, affecting not only mastication but also swallowing and speech, vital actions with direct interference in survival and sometimes subject to the influence of aging^5^. It should be noted that women of reproductive age are more vulnerable^1–9,17^. However, the reason underlying this finding is still controversial. Our study indicates that the presence of testosterone receptors in such muscles is potentially a factor of protection against temporomandibular dysfunction, given that activation of the receptors would exert an anabolic effect on muscle cells^26^. This could be the first step toward understanding the reason for the greater incidence of muscle dysfunctions of the temporomandibular joint in the female sex^2–10,12,13^.

Another important point with respect to the sexual dimorphism of the muscles of mastication is the role played by testosterone in the Masseter muscle. This hormone makes it possible for the fast twitch fibers to be abundantly found in the muscle, thus reducing potential fatigue^14,15^, which is key in mastication. Therefore, hypotrophism of such a structure could result in temporomandibular dysfunction, because the force exerted during the biomechanical movements, necessary for the system to maintain a balance, depends on the masticatory muscles, or more precisely, on their fibers^22^. Perhaps a larger number of androgen receptors could sustain the trophism of the muscle, a protective physiological mechanism during the aging process. Moreover, testosterone is responsible for an increase in hemoglobin levels, in biogenesis, and in the capillary density of skeletal muscle, thus assisting in the stimulation of muscle trophism^27^.

Still, testosterone action through its binding to the receptors of the Masseter muscle would emphasize the muscle’s anabolism, engendering different contraction patterns in the genders, as has been observed in rabbit Masseter muscles, whose contraction interval is shorter in males than in females^15^. In general, the slower contraction motor unit of the males is still faster than most of the female motor units^11^. Theoretically, testosterone would protect the male sex, diminishing fatigue^14,15^, thereby leading to less muscle dysfunction. Also, testosterone promotes an increase in muscle mass as it stimulates the hypertrophy of type I and type II fibers, which are sites of protein synthesis^14,15^, and inhibits the degradation of muscle protein^16^.

In the masticatory system, the muscles which directly promote the contact between the upper and lower teeth are the so-called masticatory muscles, namely the Masseter, Temporal, Medial Pterygoid, and Lateral Pterygoid muscles. However, we included in our study the Digastric muscle due to its function during mouth opening and the attendant interference in the performance of the other muscles. Unfortunately, analysis of the lateral Pterygoid muscle was prevented owing to severe maceration during the removal process, because its small dimension and anatomical location (origin and insertion) hinder removal^19–21^. Nonetheless, it should be stressed that these muscles of interest are skeletal muscles and, consequently, a target of the hormonal action of testosterone^16^.

Furthermore, since the masticatory act is performed throughout life, there is a constant demand for the functionality of the muscles involved in mastication. With aging, hormone levels drop, and this is part of the natural physiological process. In rats, aging significantly reduces testosterone levels. Younger rats (3 months) would have 2 to 4 times higher serum testosterone levels than older ones (12 months)^25^. Hence, the bioavailability of the hormone would lessen as age advanced. The increase in androgen receptor expression in older animals might compensate for the relative deficiency of testosterone during the aging process by maintaining the performance of an activity as vital as mastication and by reducing muscle dysfunction as well.

In blood circulation, testosterone and other sex hormones are bound to binding proteins, which play an important role in regulating their transportation, distribution, metabolism, and biological activity. The most important of these proteins is SBHG (steroid hormone-binding protein); however, under certain conditions, such as obesity and malnutrition, the binding^28^ proteins undergo changes^28^. Besides, dihydrotestosterone has greater affinity for the androgen receptor than for testosterone. Nevertheless, its conversion takes place inside the target cell through 5-alpha reductase. Some antiandrogen substances, such as finasteride, may affect not only conversion, but also affinity to the receptor^27^. As our animal model was raised solely for this research, such situations were not observed, for testosterone bioavailability was not influenced by these factors, it was affected by age alone.

Androgens also act in local inflammatory processes, reducing oxidative stress and the presence of substances harmful to muscles. There have been reports of androgen receptors in mast cells near human skeletal muscles^16^, enabling anti-inflammatory action during local cell proliferation in response to the hypertrophic stimuli of muscle contraction. In masticatory muscles, this would also constitute protection.

Testosterone is also necessary for the maturation of the trigeminal nociceptive reflex of the Digastric muscle^18^. The motor neurons of the caudal nucleus of the Trigeminal nerve, through their synapses, act on the temporomandibular muscles influencing their movements during mastication. In these neurons, the sex steroid hormones, specifically testosterone, promote their growth, change proliferation, or increase dendritic density, which actions are reflected in nociceptive responses. Underlying these testosterone effects is the activation of the androgen receptors identified in neurons and in trigeminal afferent fibers^18^. The effects are also a reflection of testosterone protection against the symptoms of temporomandibular dysfunction.

Many studies seek to determine the action of sex steroid receptors in temporomandibular joint structures, such as superficial structures, joint disc, or ligaments, to clarify the reasons for the female prevalence in temporomandibular dysfunction^6,12,13^. The presence of androgen receptors in masticatory muscles could form a line of research. In this sense, our study points to a larger number of androgen receptors mainly in the Masseter muscle, which is essential in mastication and may be involved in temporomandibular dysfunction.

## Conclusion

Testosterone receptor expression was highest in old male rats. This fact – if the necessary cautionary measures are taken in generalizing from animal studies to human reality – could indicate age- and sex-related hormonal influence on temporomandibular muscle dysfunction. Further studies, however, are necessary to strengthen this hypothesis.
